# Impact of Drip Irrigation and Nitrogen Fertilization on Soil Microbial Diversity of Spring Maize

**DOI:** 10.3390/plants11233206

**Published:** 2022-11-23

**Authors:** Sarula, Hengshan Yang, Ruifu Zhang, Yuanyuan Li, Fanhao Meng, Jinhui Ma

**Affiliations:** 1 College of Agronomy, Inner Mongolia Minzu University, Tongliao 028000, China; 2 Research Center of Forage Crop Engineering Technology, Tongliao 028042, China

**Keywords:** water-saving, nitrogen reduction, shallow buried dropper, soil, bacteria, fungi, microbial diversity

## Abstract

Given the shortage of water resources and excessive application of nitrogen fertilizers in irrigated areas, we explored the effect of water–nitrogen coupling on soil microbial diversity in maize fields irrigated using shallow buried droppers. A field experiment (split-plot design) was used with irrigation amounts set at 40%, 50%, and 60% of the conventional amount; furthermore, 13 water and nitrogen coupling treatments were designed. The secondary area was the nitrogen application level, corresponding to 50%, 70%, and the original conventional application amounts. The results showed that the effect of irrigation amount on bacterial community composition was greater than that of nitrogen, whereas the effect of nitrogen on fungi was greater than that on bacteria. No significant difference was detected in the α diversity index or species richness of bacteria and fungi. Available phosphorus and organic carbon contents significantly correlated with the community structure of soil bacteria (*p* < 0.05). The relative abundances of bacteria and fungi were stable with the decrease of nitrogen application rate at the irrigation rate of 2000 m^3^ ha^−1^. With the decrease of irrigation amount, the relative abundance of bacteria and fungi was stable under the treatment of 210 kg ha^−1^ nitrogen fertilizer. Moreover, the relative abundance of nitrogen-fixing bacteria related to the nitrogen cycle was increased by irrigation of 2000 m^3^ ha^−1^ and nitrogen application of 210 kg ha^−1^. Moderate reduction of subsequent N supply should be as a prior soil management option in a high N input agroecosystem.

## 1. Introduction

Nitrogen fertilization is important for optimizing short-term crop yield [1], as nitrogen availability is a key factor controlling soil carbon cycling and storage [2]. The organic carbon storage rate of soil in maize fields under continuous cropping increases with the increase in the nitrogen application amount, reaching a maximum value at the optimal nitrogen application level [1]. However, excessive nitrogen application has been shown to affect the composition and quantity of organic carbon and nitrogen in the drylands of temperate continental climate, thereby altering soil nitrogen availability [3]. Maize yield response to nitrogen is related to the amount of irrigation [4]. Under a water deficit, the amount of nitrogen required for maximum yield decreases [4].

Water is essential for agriculture in many world regions and for achieving sustainability in production systems. Water scarcity is seriously affecting agricultural production, especially in arid and semi-arid areas. Compared with the traditional method of rough broadcast fertilizer under strip-tillage and hop irrigation by flooding the inter-row, drip irrigation can deliver fertilizers and water to the roots of crops more accurately and reduce the amount of fertilizer required, minimizing environmental risks due to deep leakage of soil moisture and nutrients [5]. The drip irrigation method is known to have better water use as well as fertilizer use efficiency as compared to other methods of irrigation, particularly if poor quality water is to be used. Shallow buried droppers are a kind of subsurface drip irrigation technology in which the drip pipe is buried 3–5 cm above the surface of the ground without a film covering. Shallow burial drip irrigation with low irrigation frequency increases the moisture volume of soil; however, it does not significantly increase the amount of water seepage under plant roots [6]. Nevertheless, it is a new water-saving and high-yield technology. It was selected in 2021 as the principal agricultural technology in China, with a cumulative promotion area of more than 2 M ha. It can also reduce water consumption and improve the utilization efficiency of irrigation water [7]. Additionally, it can compensate for the lack of drainage facilities and solve the problems faced by salt-affected farmlands [8] as it does not add to soil salinity [6]. Maize yields show no negative effects at drip irrigation levels of up to 75% compared with full irrigation [9].

Further comparison with full irrigation has revealed that drip irrigation saves up to 25% of water [9], whereas fractional nitrogen application improves the efficiency of nitrogen fertilizer and irrigation water usage [10,11]. The full irrigation with the subsurface drip irrigation system maximizes potato yield but decreases irrigation water use efficiency, whereas integration of the subsurface drip irrigation system with deficit irrigation is effective in improving water productivity due to less water being consumed, allowing these practices to be used under scarce water conditions [12]. Maximum nitrogen use efficiency was obtained with good quality irrigation treatment [13]. Deficit drip irrigation based on crop evapotranspiration and precipitation forecast is beneficial to improve crop water use efficiency and maintain high grain yield in semi-arid and semi-humid regions [14]. While the effects of irrigation treatments on average dry matter contents of the lines were not found to be significant, significant differences were observed in water use efficiency and kernel yield of dent corn lines [15]. Calcium chloride activated carbonized biogas fermentation residues can adsorb phosphorus. Phosphorus captured by this sorbent is readily available for plant nutrition [16].Wood biochar shows excellent results in increasing the amount of plant-available water content in soil and appears to be an excellent tool for recycling nutrients (especially into plant-available forms of phosphorus and nitrogen) [17].Therefore, calcium chloride activated carbonization of fermentation residues and biochar [18] were used as novel fertilizers in drip irrigation. Earlier studies have focused on water and nutrient usage efficiency through microbial diversity indicators to better understand the future direction of soil function [19]. Soil microorganisms are an important part of farmland ecosystems as well as the driving force for the decomposition, transformation, and circulation of organic matter. Soil nitrogen availability may be mediated by changes in microbial community composition to modulate the microbial response to precipitation changes [20], affecting microbial biomass dynamics [2]. Furthermore, the microbial and biochemical properties of soil differ under different nitrogen concentrations and water conditions [21]. Excessive nitrogen application has been shown to significantly increase the nitrogen content of soil microbial biomass in the 0–20 cm and 20–40 cm soil layers of northwest drylands [3].

Furthermore, excessive application of chemical fertilizers can deteriorate the microbial properties and biochemical functions of the soil [22]. Moreover, microbial communities have a higher alpha diversity under nitrogen deficiency stress [23]. N has significant, strong effects on bacterial, fungal, and functional community compositions; the relative abundance of most bacterial nitrogen cycling genes is increased or unaffected by N. In contrast, N decreases or does not change the expression of most bacterial carbon degradation genes [24]. Bacteria and fungi respond differently to organic and inorganic fertilizers [25]. Actinomycetes and Gram-positive bacteria can utilize soluble organic nitrogen more efficiently, whereas nitrogen fertilizers are more efficiently utilized by Gram-negative bacteria [26]. Fertilization changes, especially long-term, can considerably affect the community structure, population, and function. Similarly, soil moisture conditions affect organic matter composition, aeration status, and microbial activity, influencing the mineralization and organic carbon mineralization rate [21]. Soil moisture correlates with the C and N metabolic potential of the bacterial and archaeal community. Moderate deficit irrigation increases water productivity without affecting microbial communities [27]. In addition, deficit irrigation increases the extracellular polysaccharide to microbial biomass ratios [28]. Soil microorganisms provide essential nutrients for crop growth. Their biomass and diversity are sensitive to changes in soil nutrients, organic matter, and pH and are potential indicators of soil quality. Neither excessive nor insufficient nitrogen application and irrigation amounts are conducive to the reproduction of aerobic voluntary nitrogen-fixing bacteria [29]. The increased precipitation in typical semi-arid areas of inner Mongolia grassland plays an important role in enhancing microbial activity. Increasing precipitation can alleviate the effect of nitrogen on microbial community composition [30]. Water replenishment and nitrogen application significantly affect the activity of soil microbial communities in artificial grasslands [31]. Watering alters the stress tolerance of desert scrub soil microorganisms, and fertilization alters the nutrient/oligotrophic properties of the microbial community [32]. Carbon source and nitrogen source have the same biodegradability [33]. Fertilization changes the availability of nutrients in agricultural raw materials. The combined effects of irrigation and fertilization on farmland soil bacterial and fungal communities are less studied.

However, the effect of water–nitrogen coupling on soil microbial diversity and activity is complex. Although knowledge regarding the effects of a single factor on soil microbial communities is growing rapidly, not much is known about the interactive effects of these two environmental change factors. In this study, high-throughput sequencing was performed using the Illumina Mi Seq platform to understand the composition and species richness of bacterial and fungal communities. We aimed to reveal the composition characteristics and changes in functional microbial flora in soil under the combined effect of different water conditions and nitrogen concentrations during shallow burial drip irrigation. Understanding the relationship between environmental factors might help clarify the coupling effect of the microbial and chemical properties of soil, providing a theoretical basis for constructing a high-yield and high-quality ecological environment.

This paper utilizes lots of acronym names, all the abbreviations used and their full names to create a list (Table 1).

## 2. Results

### 2.1. Bacterial and Fungal Community Composition in Soil with Water and Nitrogen Reduction under Shallow Burial Drip Irrigation

**Figure 1:**
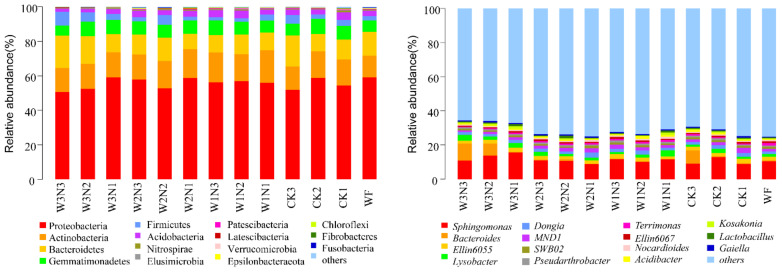
Differences in the taxonomic composition of soil bacteria at the phyla and genus levels.

**Figure 2:**
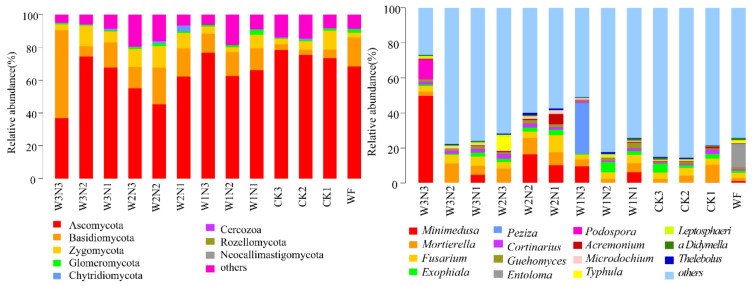
Differences in the taxonomic composition of soil fungi at the phyla and genus levels.

The sequencing (Table 2) results showed that the soil bacteria belonged to 35 phyla, 96 classes, 234 orders, 415 families, and 939 genera (Figure 1), mainly Proteobacteria, Actinobacteria, Pseudobacterium, and Bacteroidetes, in which Proteobacteria had the highest abundance. At the genus level, *Sphingomonas* and *Bacteroides* were the most abundant. The sample soil fungi belonged to 10 phyla, 29 classes, 76 orders, 137 families, and 207 genera. Figure 2 shows that the sample soil fungi were mainly distributed in the phyla Ascomycota, Basidiomycota, and Zygomycota and belonged to the genera *Minimedusa*, *Mortierella*, *Fusarium*, *Exophiala*, and *Peziza*.

**Figure 3:**
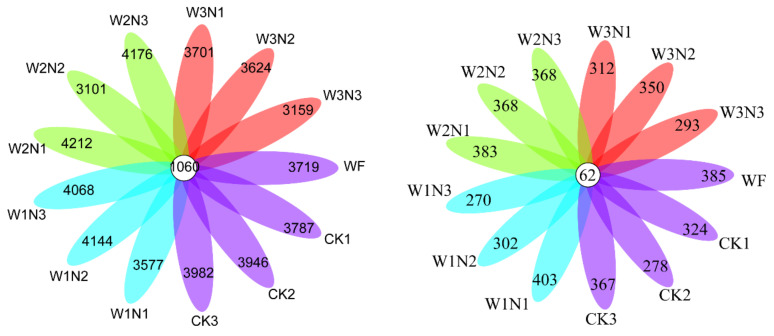
Statistics on the numbers of bacteria and fungi operating taxa.

The numbers of effective sequences obtained from soil bacteria and fungi were 54,724 and 73,178, respectively. The total number of bacterial operational taxonomic units (OTUs) in each treatment was 1060 (Figure 3), with the largest number of bacterial OTUs present in W2N1. The numbers of unique OTUs in WF, CK1, CK2, and CK3 groups were 3719, 3787, 3946, and 3982, and the number of fertilizer-free OTUs was the least under shallow burial conditions. The number of bacterial OTUs increased upon application of NPK fertilizer or K and P fertilizer. Under the same fertilization amount, the number of bacterial OTUs under conventional nitrogen fertilization and conventional border irrigation exceeded that in conventional nitrogen fertilization in shallow burial drip irrigation. At W2 and W3 levels, the number of OTUs gradually decreased with an increase in the nitrogen application amount. The number of bacterial OTUs in the N1 fertilization amount was greater than that in N2 and N3. At the W1 level, the number of bacterial OTUs in N2 was greater than in N3 and N1. Under different irrigation regimes, the number of fungal OTUs decreased with an increase in fertilization. Additionally, the number of fungal OTUs in shallow burial drip irrigation was greater than that in traditional border irrigation. The number of fungal OTUs in CK3 was lower than in WF in shallow burial without nitrogen application.

At the N1, N2, and N3 levels, W2N1, W2N2, and W2N3 had the highest number of fungal OTUs, which was higher than those of CK1, CK2, CK3, and WF. At the N1 level, the number of fungal OTUs gradually decreased with a decrease in irrigation amount, and at N2 and N3 levels, the combination of fungal OTUs with W2 was the largest. Compared with CK2, W1N2, W1N3, W2N1, and W2N3 had an increased number of bacterial OTUs. Except for W1N3, the other shallow burial drip irrigation water and nitrogen reduction treatments had increased the number of fungal OTUs.

### 2.2. Correlation Analysis of Soil Properties, Maize Yield, and Microbial Community Structure

**Figure 4:**
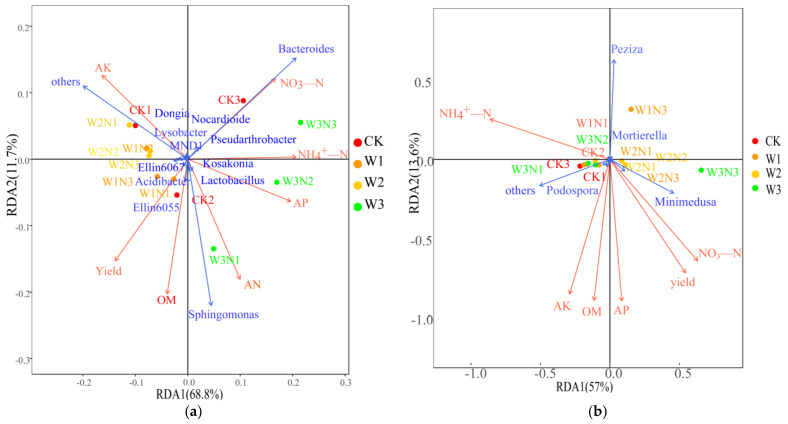
Analysis of the relationship between soil environmental factors and key microorganisms. Note: The red arrows represent different environmental factors, and the blue arrows represent the various microbial genera. The angle between species and environmental factors represents the positive and negative correlation between them (acute angle: positive correlation; obtuse angle: negative correlation; right angle: no correlation). (**a**): the analysis of the relationship between soil environmental factors and soil bacteria; (**b**): the analysis of the relationship between soil environmental factors and soil fungi.

*Sphingomonas* was positively correlated with available phosphorus (AP), alkali-hydrolyzed nitrogen (AN), organic matter (OM), and yield and negatively correlated with nitrate nitrogen (NO_3_-N) and available potassium (AK). *Bacteroides* was positively correlated with NO_3_-N, ammonium nitrogen (NH_4_^+^-N), and AP, and negatively correlated with AN and OM; however, no correlation was observed with AK. *Ellin6055* was positively correlated with AP, AN, and OM, and negatively correlated with AK and NO_3_-N. *Lysobacter* positively correlated with AK and NO_3_-N, and inversely correlated with AP, AN, and OM. *Minimedusa* was positively correlated with yield, NO_3_-N, AN, OM, AK, and AP and negatively correlated with NH_4_^+^-N; the correlation between *Peziza* and environmental factors and yield was opposite to that of *Minimedusa* (Figure 4).

### 2.3. Analysis of Different Species of Soil Bacteria and Fungi under Shallow Drip Irrigation

**Figure 5:**
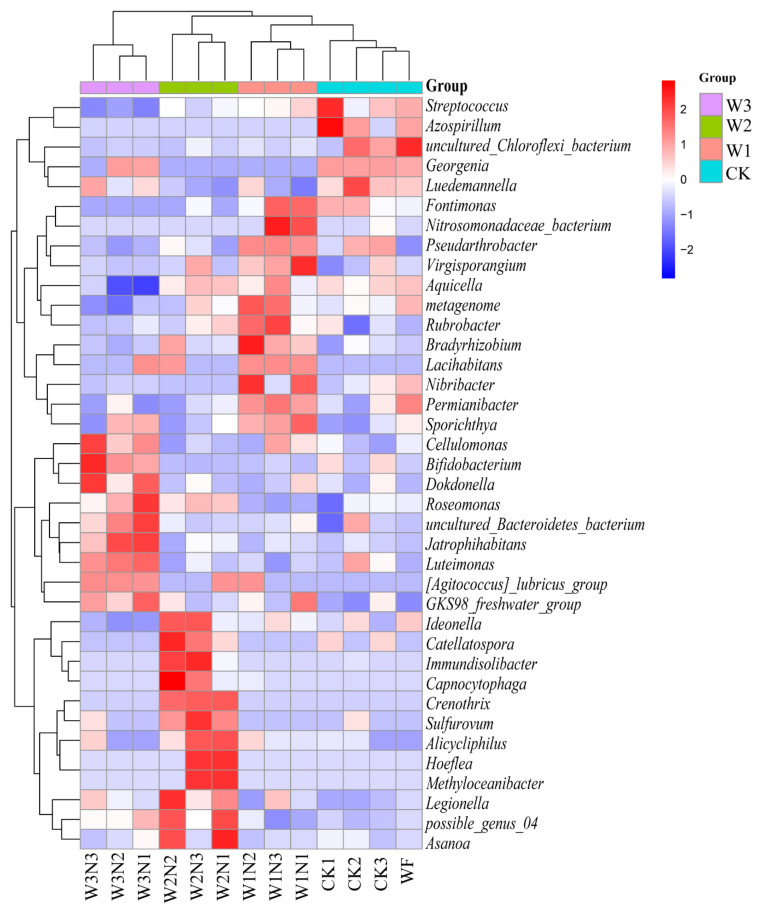
Thermal map of differential species abundance of bacteria. Note: The above picture shows the heat map at the genus level; the horizontal axis indicates the sample information, and the vertical axis is the species annotation. On the left side of the figure is the species clustering tree and the clustering branch group above indicates that the samples come from different groups. The red and blue tiles indicate higher and lower relative abundance, respectively.

The horizontal clustering results showed that *Streptococcus*, *Azospirillum*, uncultured_Chloroflexi_bacterium, *Georgenia*, and *Luedemannella* were close in terms of distance and had short branch length. *Fontimonas*, uncultured_Nitrosomonadaceae_bacterium, *Pseudarthrobacter*, *Virgisporangium*, *Aquicella*, *Rubrobacter*, *Bradyrhizobium*, *Lacihabitans*, *Nibribacter*, *Permianibacter*, and *Sporichthya* were close in terms of distance and had short branch lengths. *Cellulomonas*, *Bifidobacterium*, *Dokdonella*, *Roseomonas*, uncultured_Bacteroidetes_bacterium, *Jatrophihabitans*, *Luteimonas*, [*Agitococcus*]_*lubricus*_group, and GKS98_freshwater_group were close in terms of distance and had short branch lengths. *Ideonella*, *Catellatospora*, *Immundisolibacter*, *Capnocytophaga*, *Crenothrix*, *Sulfurovum*, *Alicycliphilus*, *Hoeflea*, *Methyloceanibacter*, *Legionella*, possible_genus_04, and *Asanoa* were close in terms of distance and had short branch length, indicating that these species have similar compositions among samples (Figure 5).

Based on the vertical clustering results, the following were classified into one category because they were close in terms of distance and had short branch lengths: W3N3, W3N2, and W3N1; W2N3, W2N2, and W2N1; W1N3, W1N2, and W1N1; CK1, CK2, CK3, and WF. This result indicated that the composition and abundance of these samples were similar, and soil bacteria were more sensitive to the amount of irrigation than to the amount of nitrogen fertilizer. Moreover, the composition and abundance of these samples were similar, and the response of soil bacteria was more sensitive to the amount of irrigation than the amount of nitrogen fertilizer.

At the W3 level, with a decrease in the nitrogen application amount, the relative abundance of *Jatrophihabitans* and uncultured_Bacteroidetes_bacterium increased, whereas the relative abundance of *Dokdonella*, *Cellulomonas*, and *Bifidobacterium* decreased. The relatively stable bacterial groups were Luteimonas and [*Agitococcus*]_*lubricus*_group, *Sulfurovum*, *Alicycliphilus*, *Legionella*, *Luedemannella*, and *Roseomonas*, which can efficiently degrade several organophosphorus pesticides [34], and were newly emerged in W3N3. *Sporichthya*, *Lacihabitans*, and *Georgenia*, which can degrade several organophosphorus pesticides, disappeared. At the W2 level, with a decrease in the nitrogen application amount, the relative abundance of *Sulfurovum*, *Immundisolibacter*, *Roseomonas*, and *Ideonella* decreased, and that of *Capnocytophaga* increased. The relative abundance of *Crenothrix* was relatively stable, and those of *Legionella* and *Catellatospora* first decreased and then increased. Metagenome and *Virgisporangium* newly emerged in W2N3, whereas *Asanoa* and *Legionella* colonies disappeared. At the W1 level, with the decrease in the nitrogen application amount, the relative abundances of *Permianibacter*, *Rubrobacter*, metagenome, *Fontimonas*, and uncultured_*Nitrosomonadaceae*_bacterium decreased. The relative abundance of *Sporichthya* and *Virgisporangium* increased, and that of *Lacihabitans* and *Pseudarthrobacter* was relatively stable. *Legionella*, *Ideonella*, *Dokdonella*, *Aquicella*, and *Streptococcus* newly emerged in W1N3, whereas *Rubrobacter* and *Nibribacter* flora disappeared. [*Agitococcus*]_*lubricus*_group and *Luedemannella* newly appeared in W1N2.

**Figure 6:**
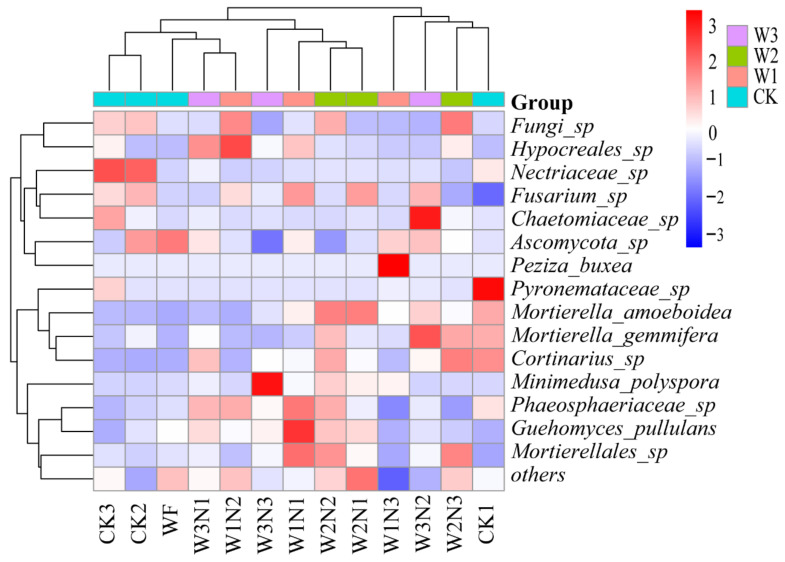
Thermal map of differential species abundance of fungi. Note: The above picture shows the heat map at the genus level; the horizontal axis indicates the sample information, and the vertical axis is the species annotation. On the left side of the figure is the species clustering tree and the clustering branch group above indicates that the samples come from different groups. The red and blue tiles indicate higher and lower relative abundance, respectively.

The horizontal clustering results showed that fungi_sp, *Hypocreales*_sp, *Nectriaceae*_sp, *Fusarium*_sp, *Chaetomiaceae*_sp, Ascomycota_sp, and *Peziza_buxea* were close in terms of distance and had short branch lengths. *Pyronemataceae*_sp, *Mortierella*_*amoeboidea*, *Mortierella*_*gemmifera*, and *Cortinarius*_sp were close and had short branch lengths. *Guehomyces_pullulans* and *Mortierellales*_sp were close in terms of distance with short branch lengths, indicating that the soil fungal compositions of these species were similar among treatments. According to the longitudinal clustering results, CK2, CK3, WF, W3N1, and W1N2 were close in terms of distance, had short branch length, and were classified into one category. W3N3, W1N1, W2N2, and W2N1 were close in terms of distance, had short branch length, and were classified into one category. W1N3, W3N2, W2N3, and CK1 were close in terms of distance, had short branch length, and were classified into one category, indicating that these treatments yielded similar soil fungal compositions and relative abundance profiles (Figure 6).

**Figure 7:**
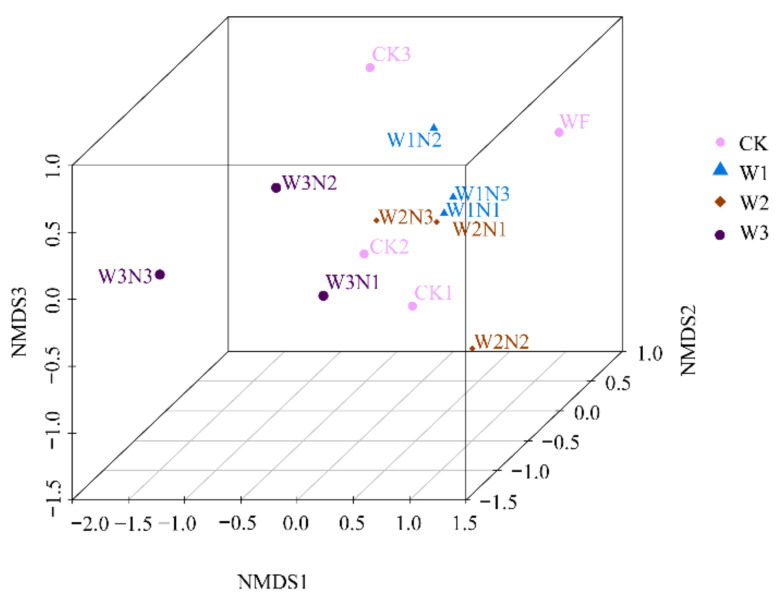
Beta diversity analysis of each treatment. Note: The horizontal and vertical axes represent the two eigenvalues that can best reflect the variance. Each point in the graph represents a sample. Similar samples are clustered together, and if the differences between samples are large, they will be farther apart in the graph.

Non-metric multi-dimensional scaling method (NMDS) analysis showed that the distances between WF and CK1, as well as CK2 and CK3, were relatively long; the distances between CK1 and CK2, as well as W1N1, W1N3, and W2N1 were relatively shorter (Figure 7).

There was no significant difference in the Chao1 index of bacteria and fungi among the groups, indicating no difference in their richness. The Shannon index of bacteria in the W3 group was significantly lower than that in W1, W2, and CK, and the Shannon index of fungi in the W3 group was significantly lower than that in W2; however, there was no significant difference between W1, W3, and CK. This indicated that the bacterial diversity of samples in group W3 was lower than that in other groups, and the fungal diversity of samples in the W3 group was lower than that in the W2 group (Table 3).

**Figure 8:**
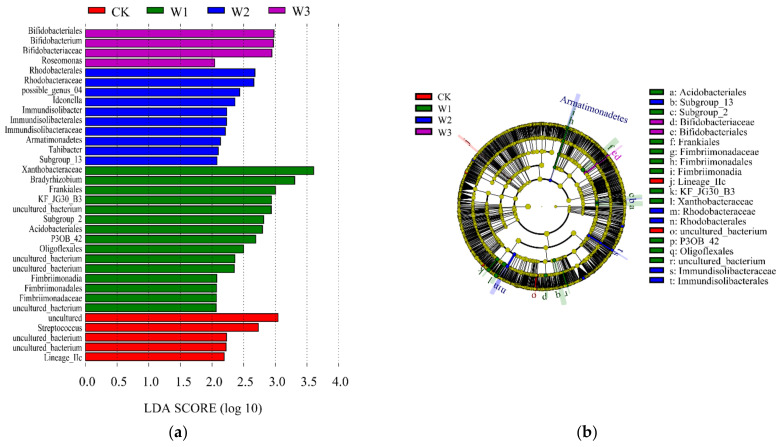
Analysis of bacteria difference between groups. Note: (**a**)–Differential species score chart; different colors indicate distinct groups; the bars indicate the relative abundance in the groups. (**b**)–Annotated branch diagram of different species; distinct colors represent different groups, red nodes represent significantly different species with relatively high abundance, and green nodes represent relatively high differences in abundance. Significant species: the yellow nodes represent species with no significant difference between groups. The diameter of the node is proportional to the relative abundance. Each layer of nodes represents phylum/class/order/family/genus from the inside to the outside. Annotations of the layer species labels indicate the phylum/class/order/family/genus from the inside to the outside; the species names represented by the alphabets in the figure are shown in the legend on the right.

According to the linear discriminant analysis effect size (LEfSe) analysis of the four groups of samples, bacteria with LDA values greater than 2.5 were screened. Differences were observed in the species with higher relative abundance in CK, W1, W2, and W3 groups. The different bacterial groups were mainly from uncultured_bacterium, *Streptococcus*, *Xanthobacteraceae*, *Bradyrhizobium*, *Rhodobacteraceae*, Rhodobacterales, *Bifidobacteriaceae*, *Bifidobacterium*, and Bifidobacteriales. The species significantly differed in each group (Figure 8). Lineage_IIc and uncultured_bacterium in CK; Acidobacteriales, Subgroup_2, Frankiales, *Fimbriimonadaceae*, Fimbriimonadales, Fimbriimonadia, and KF_JG30_B3 in W1; and *Xanthobacteraceae*, P3OB_42, Oligoflexales, uncultured_bacterium, Subgroup_13, *Rhodobacteraceae*, Rhodobacterales, *Immundisolibacteraceae*, Immundisolibacterales, *Bifidobacteriaceae*, and Bifidobacteriales in W3.

**Figure 9:**
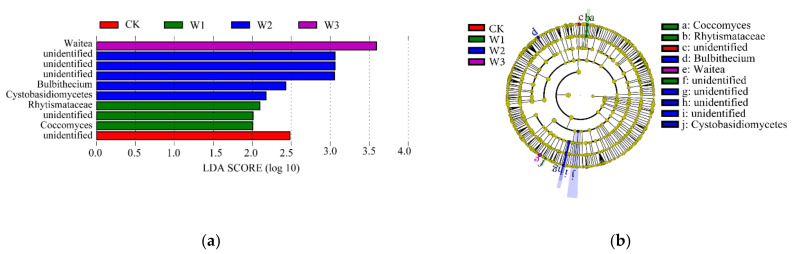
Fungus analysis of difference between groups. Note: (**a**)–Differential species score chart; different colors indicate distinct groups; the bars indicate the relative abundance in the groups. (**b**)–Annotated branch diagram of different species; distinct colors represent different groups, red nodes represent significantly different species with relatively high abundance, and green nodes represent relatively high differences in abundance. Significant species: the yellow nodes represent species with no significant difference between groups. The diameter of the node is proportional to the relative abundance. Each layer of nodes represents phylum/class/order/family/genus from the inside to the outside. Annotations of the layer species labels indicate the phylum/class/order/family/genus from the inside to the outside; the species names represented by the alphabets in the figure are shown in the legend on the right.

The fungal LEfSe analysis of the four groups of samples (Figure 9) screened species with LDA values greater than two. Differences were observed in the fungal diversity among CK, W1, W2, and W3 groups, and the different groups were mainly from unidentified *Rhytismataceae*, Cystobasidiomycetes, *Bulbitecium*, and *Waitea.* Moreover, there were significant intergroup differences (Figure 9).

### 2.4. Input and Output Analysis

Except W1N1, the net income from other treatments is greater than CK2 (Table 4). Shallow burial drip irrigation is characterized by saving labor, time, water and investment.

## 3. Discussion

Soil microbial characteristics are regulated by irrigation and are closely related to the cyclic transformation of soil C and N nutrients [35]. An irrigation quota of 900 m^3^·ha^−1^ in the Hetao Irrigation area was found to be the most conducive to the reproduction of bacteria and actinomycetes in saline soil, whereas a quota of 1125 m^3^·ha^−1^ was the best suited for fungal reproduction [36]. Thus, under high water content conditions, the Shannon, Ace, and Chao1 indices of the bacterial community in the vegetable fields increased significantly. With the increase in soil water content, the relative abundance of *Aeromonas* and *Flavobacterium* also increases [37]. In the present study, under high nitrogen conditions, the relative abundance of *Bacteroides*, *Lysobacter*, and *Ellin6055* increased with an increase in irrigation amount, and the nitrogen reduction treatment of shallow burial drip irrigation decreased the relative abundance of *Sphingomonas*. In the study by Yan et al. [38], ammonification bacteria significantly increased, and N-fixing bacteria significantly decreased with precipitation reduction. Dinev et al. [39] showed that fertilization has the strongest multiplication effect on the number of aerobic mesophilic microorganisms in the soil, whereas irrigation has no statistically significant impact. In the study by Jiao et al. [40], with the increase in soil moisture, the dominant bacterial species shifted from Actinobacteria to Alphaproteobacteria and Acidobacteria. The same pattern was observed in the low nitrogen (N1) group in this study; however, in the high (N3) and middle nitrogen (N2) groups, the trend was different; that is, the relative abundance of Acidobacteria decreased with the increase of irrigation amount in the high nitrogen (N3) group and was more stable in the medium nitrogen (N3) group; low soil moisture content significantly reduced enzyme activity and bacterial richness [41]. The composition of the bacterial community varied with soil moisture, but the fungal community was more resistant to water stress and acquired labile C more efficiently under low moisture levels [42]. In the study by Jiao et al. [40], there was no significant difference in Shannon diversity, relative abundance, and community structure of fungi under different soil water conditions. In addition, bacteria were more affected by stepwise wetting processes than fungi, which is consistent with the results of this study.

Fertilization affects the function and composition of the microbial community [43]. When the optimum nitrogen application amount for maize in the Hetao Irrigation Area of Inner Mongolia was 280.11 k·ha^−1^, soil microorganisms peaked, and the increasing effect was the most significant [44]. If N is present in conventional bare-field maize fertilizers, the effects of P and K on soil microorganisms are not evident [45], and a single application of nitrogen and phosphorus in maize fields in black soil areas cannot improve the diversity, uniformity, and soil microbial communities of soil bacteria and fungi. However, the composition of soil bacterial and fungal communities changed, with changes in fungal communities being more significant [46]. N fertilization increased the relative abundance of the typical copiotrophic bacterial taxa, Alphaproteobacteria, but reduced that of the oligotrophic group, Acidobacteria. N fertilization stimulated most C transformation and N cycling processes [47]. In this study, the relative abundance of Acidobacteria decreased with the increase of the N application rate under high irrigation (W3) but increased with increasing the N application rate under low irrigation (W1). Yang et al. [48] selected an eco-friendly nitrogen (N) application level for sugarcane production, showing that soil bacterial richness could be significantly promoted by the medium (482 kg ha^−1^) and high nitrogen (964 kg ha^−1^) treatments. However, soil bacterial diversity could not be significantly improved. On the contrary, soil bacterial diversity and richness could be improved by low nitrogen (96 kg ha^−1^) treatment. N-fertilized soil to reduce subsequent N supply (RCN) did not affect bacterial diversity, whereas RCN altered the community structure by enriching beneficial taxa, such as Actinobacteria (at the phylum level), and Streptomyces, Kribbella, Gaiella, Gemmatimonas (at the genus level), etc. The complexity and connectivity of the bacterial co-occurrence network were enhanced by RCN [49]. In this study, W2N2 treatment enriched Glomeromycota, nitrogen-fixing bacteria Rhodanobacter and Bradyrhizobium, which make up the arbuscular mycorrhiza of terrestrial plants. In the present study, the effect of nitrogen on fungi also exceeded that on bacteria.

Fertilizer reduction treatment can increase the scale of fungal ecological network and community interaction, with a relatively close synergistic relationship between fungal communities [50]. A single application of chemical fertilizer can significantly increase soil microbial biomass carbon [51]. The response of soil microorganisms to water-saving and nitrogen-reducing agricultural measures is related to the complex interaction between water and nitrogen as well as between various groups in the food web [52]. The application of P and K in maize fields promotes the number of soil bacteria, and the application of inorganic fertilizers changes the community structure of soil microorganisms [53]. A single application of nitrogen fertilizer, co-application of nitrogen-potassium-phosphorus fertilizer, and co-application of potassium and phosphorus fertilizers increase the relative abundance of soil actinomycetes and fungi during the maize growth period [53]. In the present study, the number of bacterial OTUs in shallow burial drip irrigation without fertilizer was the least, and the application of nitrogen, phosphorus, and potassium fertilizers or the application of potassium and phosphorus fertilizers both increased the number of bacterial OTUs. The application of phosphorus and potassium fertilizers increased the relative abundance of Bacteroidetes and decreased the relative abundance of Proteobacteria. The relative abundance of Actinobacteria and Gemmatimonadetes did not change, that of Proteobacteria and Gemmatimonadetes increased, and that of Bacteroidetes decreased after nitrogen application. In this study, soil fungi were mainly distributed in Ascomycota, Basidiomycota, and Zygomycota. The application of phosphorus and potassium fertilizers increased the relative abundance of Ascomycota and decreased that of Basidiomycota but did not affect the relative abundance of Zygomycota. Nitrogen application increased the relative abundance of Basidiomycota and Zygomycota and decreased that of Ascomycota. NPK fertilizers increased the relative abundance of Ascomycota and Zygomycota but decreased that of Basidiomycota. It was observed that the effect of phosphorus and potassium fertilizers on Ascomycota and Basidiomycota was greater than that of nitrogen fertilizers, and the effect of nitrogen fertilizers on the relative abundance of Zygomycota was greater than that of phosphorus and potassium fertilizers. Optimal nitrogen application reduced the fungal richness and diversity, decreasing bacterial richness [23]. The relative abundances of nitrogen-fixing bacteria *Rhodanobacter* and *Bradyrhizobium* related to the nitrogen cycle in the optimized nitrogen application treatment were higher than those in conventional nitrogen application treatment [54]. In the present study, different species in the 50% and 60% conventional irrigation groups and others also belonged to these two genera. Excessive or insufficient nitrogen application and irrigation amounts were not conducive to the reproduction of aerobic autogenic nitrogen-fixing bacteria.

The effect of the nitrogen application amount on the number of anaerobic authigenic nitrogen-fixing bacteria is irregular [29]. Only one genus, *Luedemannella*, has been identified under high water and high nitrogen treatment [55], and only this genus appeared in the N3 assemblage in the present study. Our previous study showed that optimal nitrogen topdressing (70% constant nitrogen topdressing) under shallow burial drip irrigation reduces carbon and nitrogen emissions from farmland ecosystems in the West Liaohe Plain, improving the carbon efficiency and effective use of nitrogen inputs [56]. Under natural precipitation, when the irrigation amount is 1958.40–2228.00 m^3^·ha^−1^, the nitrogen application amount is 209.34–275.70 kg·ha^−1^, the density is 67,350–78,150 plants·ha^−1^, and the yield can reach 12,000.00–12,716.82 kg·ha^−1^ [57]. There are numerous potential bacterial candidates that could be recruited to assist plants during water-limiting conditions [58]. In the present study, with a decrease in the nitrogen application amount, compared with the irrigation amounts of 1600 and 2400 m^3^·ha^−1^, the relative abundance of Actinobacteria and Gemmatimonadetes under the irrigation amount of 2000 m^3^·ha^−1^ was more stable under high nitrogen conditions. The relative abundances of Proteobacteria, Firmicutes, and Actinobacteria did not change with low-nitrogen treatment, and the relative abundance of Basidiomycota, Zygomycota, and Glomeromycota bacteria was the highest in W2N2 (210 kg·ha^−1^ nitrogen application) treatment. The relative abundance of *Minimedusa*, *Mortierella*, *Guehomyces*, and *Thelebolus* fungi was the lowest in W2N2; bacteria and fungi had strong antagonistic effects in topsoil. Competition and environmental filtration both affect the abundance, composition, and encoding of gene functions of bacterial and fungal communities. Of note, soil bacteria are more resistant to mineral fertilization interference than fungi [43]. Moreover, the interactions between organisms can change the microbial community [59].

Soil water and nitrogen content are the main drivers of microbial community structure [60]. Previous studies have shown that high water and normal nitrogen treatments render the bacterial community more uniform in greenhouse soil; however, they do not improve diversity [61]. With increased precipitation and nitrogen addition, the relative abundance of fungi significantly decreases [30]. Changes in the soil moisture content affect the richness of the soil fungal community in the desert steppe, whereas water and nitrogen treatments exert no notable effect on fungal community diversity [34]. Nitrogen addition weakened the effect of water addition in terms of soil bacterial diversity and community stability, and did not have an interactive influence [62]. Research by Guo et al. [63] shows that N addition, reduced precipitation and their combined effect significantly altered the soil fungal community composition. Soil microbial biomass and composition were more strongly affected by nitrogen fertilization compared with water regime. Nitrogen fertilization increased soil microbial biomass and altered soil microbial community composition, especially under low soil water availability [64]. In the present study, higher water volume irrigation in water-saving irrigation decreased soil bacterial and fungal diversity. The drip irrigation quota can affect the response of soil microorganisms to nitrogen. At 50% and 60% conventional irrigation amounts, the relative abundance of Actinobacteria was stable despite the decrease in the nitrogen application amount. In comparison, the relative abundance of Actinobacteria decreased at the 40% conventional irrigation amount. The relative abundance of Gemmatimonadetes increased with the decrease in the nitrogen application amount under the 60% conventional irrigation amount, whereas the relative abundance of Gemmatimonadetes was stable under the 50% conventional irrigation amount but decreased under the 40% conventional irrigation amount. With decrease in the nitrogen application amount, the relative abundance of Proteobacteria increased at 60% but was stable at the 40% conventional irrigation amount. Furthermore, with a decreased nitrogen application amount, the relative abundance of Bacteroidetes was relatively higher. The abundance decreased and exceeded that in N1 and N3 at the 40% and 50% conventional irrigation amounts.

Previous studies have shown that the significant spatial distribution of soil nutrients affects the composition of microbial communities [65]. The relative abundance of soil bacteria and fungi may be related to changes in soil chemical composition [66]. Liu et al. [67] showed that Zygomycota, Glomeromycota, and Chytridiomycota possessed strongly positive associations with available potassium and available phosphate, whereas Ascomycota showed a strong negative association. Plant growth-promoting bacteria, such as the genus *Sphingomonas* and families Rhizobiaceae and Micrococcaceae, are potentially associated with soil quality [68]. The total bacterial and diazotrophic population significantly positively correlated with the available NPK and organic carbon in the soil at each growth stage [69]. In the present study, the relative abundance of *Sphingomonas*, *Bacteroides*, *Ellin6055*, and *Minimedusa* was positively correlated with AP. The relative abundance of *Sphingomonas* and *Minimedusa* positively correlated with yield. The relative abundance of *Sphingomonas*, Ellin6055, and *Peziza* inversely correlated with NO_3_-N. This could be attributed to the complex effects of environmental disturbances and plants on soil microbial communities. In different studies, soil nutrients received varying degrees of disturbance, which in turn, differentially affected the composition of microbial communities; hence, the correlation between microorganisms and soil nutrients was different.

In this study, the effect of irrigation amount on bacterial community composition was greater than that of nitrogen, whereas the effect of nitrogen on fungi exceeded that on bacteria. The relative abundance of Bacteroidetes and Firmicutes decreased in the irrigation volume of 2400 m^3^·ha^−1^, and the relative abundance of Glomeromycota, which can constitute arbuscular mycorrhizae of terrestrial plants, decreased. Chytridiomycota disappeared in the irrigation volume of 1600 m^3^·ha^−1^. The irrigation volume of 2000 m^3^·ha^−1^ and the nitrogen application amount of 210 kg·ha^−1^ enhanced the ability to maintain the balance of the soil microbial community. The soil water distribution formed by shallow burial drip irrigation changes the nitrogen cycle and microbial composition, affecting the biomass of nitrifying bacteria and denitrifying bacteria. Thus, the interaction among “root system–soil–microbes” and shallow burial drip irrigation root zone environment is conducive to enhancing the interaction of “roots–soil–microbes” [70], thereby improving the carbon efficiency and effective use of nitrogen input, ultimately increasing maize yield. Under water-saving and nitrogen-reducing drip irrigation, the relationships between dominant flora and the synergistic mode of soil fungal and bacterial communities warrant further research.

The water-saving and economic benefits of shallow-buried drip irrigation were improved significantly, and the water use efficiency and benefits were increased. In the aspect of ecological benefit, after the implementation of the shallow-buried drip irrigation project, the irrigation water quantity is greatly reduced, the productivity of irrigation water is increased, and the groundwater depth is in a relatively stable state, which ensures the ecological benefit. Considering water-saving, economic and ecological benefits, the comprehensive benefits of shallow-buried drip irrigation are higher than that of traditional border irrigation. The comprehensive performance of shallow-buried drip irrigation planting pattern is better in the aspects of seedling protection effect, seed quality, yield per unit area, cost input, pure benefit, etc., the water-saving planting technology model of shallow-buried drip irrigation is characterized by saving labor, time, water, investment and pollution, which can replace the semi-film drip irrigation widely used at present, it is suitable to be popularized in a semi-arid area (Table 5).

In the follow-up study, soil metabolomics technology will be used to analyze the different metabolites in soil under the condition of water saving and nitrogen reduction, and combined microbial-metabolite analysis will be used, the relationship between soil microorganism, soil metabolite and plant will be further verified.

## 4. Conclusions

In this study, we analyzed the effects of drip irrigation and nitrogen fertilization on the soil microbial diversity of spring maize. The results showed that the relative abundance of Proteobacteria and Ascomycota decreased, that of Bacteroidetes, Firmicutes, and Basidiomycota increased, while the relative abundance of Glomeromycota, which can form arbuscular mycorrhizal fungi in terrestrial plants, increased under soils treated with shallow drip irrigation and nitrogen reduction. However, the relative abundance of Actinobacteria, Gemmatimonadetes, and Acidobacteria was unaffected by the above treatment.

The effect of irrigation amount on the bacterial community exceeded that of nitrogen, and the effect of nitrogen on fungi was greater than that on bacteria. The relative abundances of bacteria and fungi were relatively stable with the decrease of nitrogen application rate at the irrigation rate of 2000 m^3^ ha^−1^. With the decrease in irrigation amount, the relative abundance of bacteria and fungi was also relatively stable under the treatment of 210 kg ha^−1^ nitrogen fertilizer. The relative abundance of nitrogen-fixing bacteria related to the nitrogen cycle was enhanced by irrigation of 2000 m^3^ ha^−1^ and a nitrogen application rate of 210 kg ha^−1^.

## 5. Materials and Methods

### 5.1. Natural Overview of the Experimental Area and Site

The study was conducted in the Agricultural High-tech Demonstration Park (43°36′ N, 122°22′ E) of Horqin District, Tongliao City, from 2017 to 2019, at an altitude of 180 m, with an annual average temperature of 6.8 °C and active accumulated temperature of ≥10 °C of 3200 °C. The average frost-free period was 154 days, the average annual precipitation was 386.5 mm, and the soil was gray meadow soil. From 2017 to 2019, the organic mass of the plow layer (0–20 cm) in the experimental field before sowing was 18.52–19.63 g·kg^−1^. The amounts of alkaline hydrolyzable nitrogen, phosphorus, and potassium were 50.81–52.26 mg·kg^−1^, 11.35–13.20 mg·kg^−1^, and 110.83–118.69 mg·kg^−1^, respectively.

### 5.2. Experimental Design

The experimental design involved three treatment methods—conventional nitrogen application with shallow burial drip irrigation (CK1) as well as traditional border irrigation (CK2), shallow burial drip irrigation with no nitrogen fertilizer (CK3), and control with similar conditions as the latter (WF). The principal treatments, performed using a split-plot design, were determined based on the drip irrigation quota, as follows: 40% (W1), 50% (W2), and 60% (W3) conventional irrigation amounts of traditional border irrigation. The side treatment was nitrogen application, which was 50% (N1), 70% (N2), and 100% of the conventional application amount (N3). Further, we combined W1N1, W1N2, W1N3, W2N1, W2N2, W2N3, W3N1, W3N2, W3N3, CK1, CK2, CK3, and WF for a total of 13 treatments (Table 6). Urea (46% nitrogen content) was used as the fertilizer and was combined with irrigation; topdressing was applied at a ratio of 3:6:1 at the joining, big flare, and silking stages.

All treatments were conducted along with the administration of diammonium phosphate (18-46-0, 195 kg·ha^−1^) as well as potassium sulfate (0-0-50, 90 kg·ha^−1^), and were repeated thrice, yielding a total of 39 plots; the area of each plot being 72 m^2^ (10 m × 7.2 m). A 100 cm deep mulch film was buried between plot treatments to prevent mutual penetration of water and fertilizer. The tested variety was Nonghua 101, which was planted in large and small ridges (40 and 80 cm, respectively) with a density of 75,000 plants ha^−1^; Maize was planted by the integrated machine of sowing, fertilizing and spreading, and the planting pattern of large and small ridges (80 cm for large ridges, 40 cm for small ridges) was adopted, the drip irrigation belt is shallow buried 3~5 cm above the ground. The traditional border irrigation treatment used artificial furrow sprinkling. Maize seeds were sown on 2 May 2017, and 28 April 2018, and harvested on 4 October 2017, and 2 October 2018, respectively; another batch was sown on 1 May 2019, and harvested on 1 October 2019. Irrigation was performed according to soil water-holding conditions during the growth period.

### 5.3. Measurement Items and Methods

At the maturity stage, the 5-point sampling method was used to collect soil samples from the 0–20 cm soil layer. Approximately 100 g of soil was collected for each sample, placed in a sterilized Ziplock bag, and then in an ice box for further experimentation. Total DNA extraction of soil was performed in the laboratory (as explained in the following section).

#### 5.3.1. DNA Extraction and Amplification

The topsoil (0–20 cm deep) was collected using an Auger corer (between maize plants and the drip line), and the samples were stored at −80 °C for subsequent DNA extraction. Microbial DNA was extracted from 0.5 g of fresh soil collected from the topsoil using the MagPure Soil DNA LQ Kit (Guangzhou Magan Biotechnology Co., Ltd, Guangzhou, China), following the manufacturer’s instructions. DNA concentration and integrity were measured using NanoDrop 2000 (Thermo Fisher Scientific, Massachusetts USA) and agarose gel electrophoresis. Extracted DNA was stored at −20 °C until further processing. The extracted DNA was used as a template for the amplification of bacterial 16S rRNA genes and fungal internal transcribed spacer (ITS) genes using polymerase chain reaction (PCR, Bio-rad, 580BR10905, California, USA) with barcoded primers and Takara Ex Taq (Takara, Dalian, China).Amplicon(Table 7) quality was visualized using gel electrophoresis, purified with AMPure XP beads (Agencourt,), and amplified for another round of PCR. (Table 8). For bacterial diversity analysis, V3–V4 variable regions of 16S rRNA genes were amplified with universal primers 343F (5ʹ-TACGGRAGGCAGCAG-3ʹ) and 798R (5ʹ-AGGGTATCTAATCCT-3ʹ). For fungal diversity analysis, the ITS1 variable regions of ITS genes were amplified with universal primers ITS1F (5ʹ-CTTGGTCATTTAGAGGAAGTAA-3ʹ) and ITS2 (5ʹ-GCTGCGTTCTTCATCGATGC-3ʹ). The sequences of bacteria and fungi have been submitted to the NCBI Sequence Read Archive (SRA) with accession numbers PRJNA864591 (https://www.ncbi.nlm.nih.gov/sra/PRJNA864591, accessed on 1 August 2021) and PRJNA864571 (https://www.ncbi.nlm.nih.gov/sra/PRJNA864571, accessed on 1 August 2021).

#### 5.3.2. Library Construction and Sequencing

The amplicon quality was visualized using agarose gel electrophoresis. PCR (NovaSeq 6000) products were purified using AMPure XP beads (Agencourt, Beckman Coulter, Brea, CA, USA) and re-amplified using PCR. After purification with the AMPure XP beads again, the final amplicon was quantified using the Qubit dsDNA Assay Kit (Thermo Fisher Scientific, Waltham, MA, USA, Cat. No. Q32854). Concentrations were adjusted for sequencing, performed on an Illumina NovaSeq 6000 with 250 bp paired-end reads (Illumina Inc., San Diego, CA, USA; OE Biotech Company; Shanghai, China).

### 5.4. Bioinformatic Analysis

Paired-end reads were then preprocessed using the Trimmomatic software to detect and remove ambiguous bases (N). After trimming, the reads were assembled using the FLASH software; chimeric reads were detected and removed using the QIIME software (version 1.8.0). Clean reads were subjected to primer sequence removal and clustering to generate OTUs using the Vsearchi software, with a 97% similarity cutoff. A representative read of each OTU was selected using the QIIME package [73]. All representative reads were annotated and blasted against the Silva database Version 138 using the RDP classifier. The QIIME software was used for alpha and beta diversity analysis, followed by the R package for ANOVA to analyze significant differences between the groups. Furthermore, the LEfSe method was used to compare the taxonomy abundance spectrum.

### 5.5. Analysis of the Relationship between Environmental Factors and Microorganisms

Redundancy analysis (RDA) was performed using the Canoco software to determine the impact of environmental factors on the community structure. AN, AP, AK, NH_4_^+^-N, NO_3_-N, and OM levels were determined according to the methods described by [74]. AN, AP, AK, and OM were determined using the alkaline-hydrolysis diffusion method, 1 mol L^−1^ NaNO_3_ extraction method, 0.5 mol L^−1^ NaHCO_3_ extraction–molybdenum antimony anti-colorimetric method, flame photometric method and potassium dichromate oxidation-oil bath heating method, respectively. The soil NO_3_-N and NH_4_^+^-N contents were determined using a flow analyzer.

### 5.6. Input-Output Analysis Index

Input includes drip irrigation pipe, water and electricity, labor, fertilizer, seeds, land preparation and other costs. Output refers to corn income. According to the general office of the ministry of agriculture and the office of the national bureau of statistics survey plan to promote yield per unit area by building a whole county, a whole township and a whole construction system [75].

### 5.7. Statistical Analyses

SPSS 19.0 was used to analyze the data, and the LSD method was used to test the differences between treatments.

## Figures and Tables

**Table 1:** List of Abbreviations.

Acronym Name	Full Name
OTUs	Operational taxonomic units
AP	Available phosphorus
AN	Alkali-hydrolyzed nitrogen
OM	Organic matter
NO_3_-N	Nitrate nitrogen
NH_4_^+^-N	Ammonium nitrogen
AK	Available potassium
PCA	Principal component analysis
NMDS	Non-metric multi-dimensional scaling method
LDA	Linear discriminant analysis
ITS	Internal transcribed spacer
SRA	Sequence Read Archive
RDA	Redundancy analysis

**Table 2:** Valid sequence numbers.

Treatment	W3N3	W3N2	W3N1	W2N3	W2N2	W2N1	W1N3	W1N2	W1N1	CK3	CK2	CK1	WF
Bacterial valid sequence number	54,724	55,156	57,589	57,376	60,929	56,693	55,992	56,894	57,612	55,734	55,911	55,044	59,342
Fungal valid sequence number	72,913	66,870	69,560	73,178	71,577	59,803	64,356	55,218	68,760	63,732	42,389	70,678	72,998

**Table 3:** Alpha diversity index of soil bacteria and fungi in each group.

Group	Chao1 Index		Shannon Index		Simpson Index	
	Bacteria	Fungi	Bacteria	Fungi	Bacteria	Fungi
W1	6423.83 Aa	417.34 Aa	10.15 Aa	4.62 Aab	1.00 Aa	0.89 Aa
W2	6239.97 Aa	456.11 Aa	10.20 Aa	5.55 Aa	1.00 Aa	0.94 Aa
W3	6044.47 Aa	407.10 Aa	9.71 Bb	4.25 Ab	0.99 Aa	0.83 Aa
CK	6448.80 Aa	419.74 Aa	10.14 Aa	4.84 Aab	0.99 Aa	0.90 Aa

Note: Different small letters in the same column indicate significant differences at the *p* < 0.05 level among the different groups, and different capital letters in the same column indicate significant differences at the *p* < 0.01 level among different groups.

**Table 4:** Input and output analysis of shallow drip irrigation and traditional border irrigation (RMB·ha^−1^).

Treatment	Total Investment	Produce	Net Income	Difference with CK2
W1N1	7295.00	19,843.82	12,548.82	−526.18
W1N2	7475.00	21,892.44	14,417.44	1342.44
W1N3	7745.00	22,245.02	14,500.02	1425.02
W2N1	7362.50	21,839.80	14,477.30	1402.30
W2N2	7542.50	25,268.00	17,725.50	4650.50
W2N3	7812.50	24,825.54	17,013.04	3938.04
W3N1	7430.00	21,427.70	13,997.70	922.70
W3N2	7610.00	24,210.88	16,600.88	3525.88
W3N3	7880.00	24,750.70	16,870.70	3795.70
CK1	8150.00	24,500.00	16,350.00	3275.00
CK2	10,925.00	24,000.00	13,075.00	-

**Table 5:** Benefit analysis of comparative experiment in different planting patterns [71,72].

Cropping Patterns	Output Value (RMB·ha^−1^)	Input Value (RMB·ha^−1^)	Pure Benefit (RMB·ha^−1^)	Water Saving Benefit	Economic Benefit	Ecological Benefit	Comprehensive Benefit
Shallow burial drip irrigation	16,159.53	10,567.49	5592.04	0.210	0.285	0.060	0.232
Traditional border irrigation	13,288.49	12,074.99	1213.49	0.108	0.201	0.060	0.152

**Table 6:** Experimental design scheme.

Treatment	Implementation Plan	
	W (m^3^ ha^−1^)	N (kg ha^−1^)
W1N1	1600	150
W1N2	1600	210
W1N3	1600	300
W2N1	2000	150
W2N2	2000	210
W2N3	2000	300
W3N1	2400	150
W3N2	2400	210
W3N3	2400	300
CK1 (Shallow burial drip irrigation)	4000	300
CK2 (Traditional border irrigation)	4000	300
CK3 (No nitrogen fertilizer)	4000	0
WF (No fertilizer)	4000	0

**Table 7:** First Round PCR Reaction.

a. Add the Reaction System to the PCR Tubes		b. Set Up the PCR Instrument According to the Following Procedure		
Name	Volume	Temperature	Time	Cycle Number
2 × Gflex PCR Buffer	15 μL	94 °C	5 min	
5 pmol/μL primer F	1 μL	94 °C	30 s	26
5 pmol/μL primer R	1 μL	56 °C	30 s	
Template DNA	≥1 μL (50 ng)	72 °C	20 s	
Tks Gflex DNA Polymerase (1.25 U/μL)	0.6 μL	72 °C	5 min	
H_2_O	30 μL-	4 °C	hold	
Total	30 μL			

**Table 8:** Second Round PCR Reaction.

a. Add the Reaction System to the PCR Tubes		b. Set Up the PCR Instrument According to the Following Procedure		
Name	Volume	Temperature	Time	Cycle Number
2 × Gflex PCR Buffer	15 μL	94 °C	5 min	
Tks Gflex DNA Polymerase (1.25 U/μL)	0.6 μL	94 °C	30 s	7
Adapter I5	1 μL	56 °C	30 s	
Adapter I7	1 μL	72 °C	20 s	
First PCR product	≥1 μL (50 ng)	72 °C	5 min	
H_2_O	30 μL-	4 °C	hold	
Total	30 μL			

## Data Availability

The datasets for this study can be found in the [NCBI Sequence Read Archive (SRA); https://www.ncbi.nlm.nih.gov/sra/PRJNA864591 (accessed on 20 October 2022) and https://www.ncbi.nlm.nih.gov/sra/PRJNA864571 (accessed on 20 October 2022)].
